# The effects of strength-based versus deficit-based self-regulated learning strategies on students’ effort intentions

**DOI:** 10.1007/s11031-015-9488-8

**Published:** 2015-03-28

**Authors:** Djoerd Hiemstra, Nico W. Van Yperen

**Affiliations:** 1 Department of Psychology, University of Groningen, Grote Kruisstraat 2/1, 9712 TS Groningen, The Netherlands; 2 University of Groningen, Groningen, The Netherlands

**Keywords:** Self-regulated learning strategies, Professional self-development, Perceived competence, Intrinsic motivation, Effort intentions

## Abstract

In two randomized experiments, one conducted online (*n* = 174) and one in the classroom (*n* = 267), we tested the effects of two types of self-regulated learning (SRL) strategies on students’ intentions to put effort into professional development activities: *strength*-*based SRL strategies* (i.e., identifying perceived relative strengths and, subsequently, selecting professional development activities to further improve those strengths) versus *deficit*-*based SRL strategies* (i.e., identifying perceived relative shortcomings and, subsequently, selecting professional development activities to improve those shortcomings). Across both studies, analysis of variance revealed that, relative to students who used deficit-based SRL strategies, students who used strength-based SRL strategies were higher in perceived competence, intrinsic motivation, and effort intentions. Moreover, the results of multi-mediator analysis and structural equation modeling supported the hypothesis that the effect of strength-based versus deficit-based SRL strategies on students’ effort intentions was sequentially mediated by perceived competence and intrinsic motivation. Implications for the application of self-regulated learning strategies in the context of professional self-development are discussed.

## Introduction

In a knowledge-based economy it is imperative for professionals to guard their employability and to keep their skills and knowledge up to date. Accordingly, an important objective in higher professional and vocational education is to educate students to become self-regulating learners who are driven to work on their professional development throughout their career (Boekaerts 1997; Bolhuis 2003; Candy 2000; Loyens et al. 2008; Zimmerman 1990). Typically, to nurture students’ self-regulated learning (SRL) capabilities, educational institutions in western countries offer their students mentoring, tutoring, and study skills classes. In these classes, students may learn to use self-regulated learning strategies, including self-reflection and goal-selection strategies to assess their learning needs and to select professional development activities to meet those needs (Hansford et al. 2004; Jacobi 1991; Van den Boomet al. 2007). In addition, most institutions enable their students to self-select professional development activities (i.e., activities to improve their professional competencies) by offering them a choice of elective assignments, projects, minors, and internships.

In this context, an important question is which SRL strategies optimally support students’ motivation to put effort into professional development activities. In the present research, we addressed this question by examining the effects of two types of SRL strategies on students’ perceived competence, intrinsic motivation, and effort intentions: *strength*-*based SRL strategies* (i.e., identifying perceived relative strengths and, subsequently, selecting professional development activities to further improve those strengths) versus *deficit*-*based SRL strategies* (i.e., identifying individual shortcomings and, subsequently, selecting professional development activities to improve those shortcomings).

### Strength-based versus deficit-based SRL strategies

Self-regulated learning strategies refer to the self-controlled actions, such as self-evaluation, self-reflection, goal-selection, goal-setting, planning, and self-monitoring, that individuals take to acquire skills and knowledge and to optimize their learning (Sitzmann and Ely 2011; Zimmerman and Pons 1986). In higher professional and vocation education, a common practice for self-reflection and subsequent goal-selection is to review individual shortcomings and select professional development activities to improve those shortcomings. Specifically, in competency-based education, the standards that students have to meet are explicated in a competency profile. Students are then stimulated to reflect on their present level of competency relative to those standards, and to engage in professional development activities (which may be at school or on a job) to diminish the gap (Hoogveld et al. 2005; Kenkel and Peterson 2010; Lurie 2012; Pintrich 2004; Smith 2010).

Clearly, such deficit-based SRL strategies can motivate students to put effort into professional development activities. For example, control theories (Carver and Scheier 1981; Powers 1973) posit that motivated behavior results from the perception of a discrepancy between the actual situation and a standard. However, a drawback of deficit-based SRL strategies is their inherent focus on students’ shortcomings, that is, the performance dimensions on which students feel relatively incompetent. As emphasized by influential motivational theories, such as self-efficacy theory (Bandura 1997), self-determination theory (Ryan and Deci 2000), and the achievement goal approach (Elliot and Church 1997), perceived competence is an important determinant of motivation. Therefore, an exclusive focus on deficit-based SRL strategies may not be the most effective way to motivate students to put effort into professional development activities.

To address this issue, several scholars (Kluger and Nir 2010; Linley et al. 2010; Seligman et al. 2005) have proposed strength-based strategies, which entail that individuals assess their strengths, rather than their shortcomings, and select activities to further improve those strengths. Although improving shortcomings is obviously indispensable for mastering a profession, we suspect that that, to educate driven self-regulating learners, strength-based SRL strategies may make a valuable *complement* to the common deficit-based SRL strategies. Because their inherent focus on the performance dimensions on which students feel relatively competent, strength-based SRL strategies may support students’ motivation to put effort into professional development activities.

**Fig. 1:**
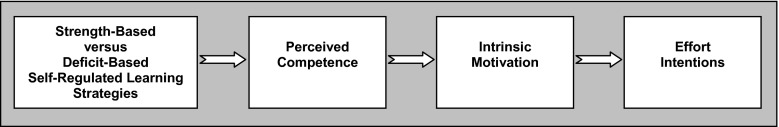
Research model

However, no research to date has examined the effects of strength-based versus deficit-based SRL strategies on students’ willingness to put effort into professional development activities. To fill this gap, we experimentally tested our research model, which posits that strength-based versus deficit-based SRL strategies positively affect effort intentions through subsequently perceived competence and intrinsic motivation (see Fig. 1).

### Toward a research model

Students who focus on improving their strengths, rather than improving their shortcomings, may feel more competent, more intrinsically motivated, and more willing to expend effort. Indeed, research indicates that working on strengths is related to various motivational concepts. For example, in a cross-sectional study, Wood et al. (2011) found a positive link between use of strengths and self-esteem. Similarly, Linley et al. (2010) found that using strengths was associated with goal progress and the fulfillment of psychological needs, including the need for competence. Furthermore, Proctor et al. (2011) found that use of strengths was associated with higher self-esteem and more self-efficacy. In addition, evidence for causal links has been obtained in a few experimental studies that compared strength-based interventions to a control group. For example, in a randomized experiment among undergraduates, Louis (2008) tested a strength-based development course against a waiting list control group and found that students in the intervention group were higher in perceived academic control. In a similar study among high school students, Austin (2005) tested a strength-based development course against a traditional health education course and found that the strength-based development course resulted in higher academic intrinsic motivation. Finally, in a study among university students, Rechter (2010) demonstrated that, relative to a traditional feedback review, a strength-based feed-forward review resulted in higher self-efficacy and stronger effort intentions. However, although the findings of these experimental studies suggest that strength-based interventions may positively affect individuals’ perceived competence, intrinsic motivation, and effort intentions, a couple of limitations should be noted. First, these studies compared broad interventions, that is, courses and reviews, which varied on multiple dimensions. Therefore, the specific causes of the reported effects cannot be determined unambiguously. Second, in these studies, strength-based interventions were not compared with deficit-based interventions. Therefore, we do not know whether strength-based interventions lead to better results than the common deficit-based interventions. Third, these experiments did not examine underlying motivational processes.

To address these limitations, in the present research, we experimentally varied the conditions on a single strength-based versus deficit-based dimension and tested the effects on both process and outcome variables. Specifically, we tested the causal effects of strength-based versus deficit-based SRL strategies on students’ effort intentions and examined the mediating effects of perceived competence and intrinsic motivation. We discuss our research model (see Fig. 1) in more detail below.

#### Perceived competence

Because strength-based SRL strategies, relative to deficit-based SRL strategies, direct students’ attention toward the positive rather than the negative aspects of their functioning, we reasoned that the effects of strength-based versus deficit-based SRL strategies on perceived competence may be similar to the effects of positive feedback versus negative feedback. Several theorists have posited that positive feedback rather than negative feedback is beneficial for learning effort, because it bolsters students’ perceived competence or self-efficacy (Bandura 1997; Ryan and Deci 2000). Indeed, research indicates that, relative to negative feedback, positive feedback enhances individuals’ self-evaluations (e.g., Baron 1988; Escartí and Guzmán 1999; Reeve and Deci 1996; Vallerand and Reid 1988; for a review, see Vallerand 1997). For example, Reeve and Deci (1996) examined the effects of (bogus) feedback on participants’ perceived competence in a puzzle-solving task. Their results showed that participants receiving negative feedback reported lower levels of perceived competence than participants receiving positive feedback. Similarly, research examining the effects of *knowledge of results* indicates that feedback on positive performances rather than feedback on negative performances enhances students’ competency perceptions and learning (e.g., Chiviacowsky and Wulf 2007; Badami et al. 2011; Saemi et al. 2012). For example, Saemi et al. (2012) found that providing students with feedback after relatively good trials on a motor learning task resulted in higher self-efficacy compared with providing feedback after weaker trials.

#### Intrinsic motivation

The effects of perceived competence on intrinsic motivation have been articulated in several theories. For example, both effectance motivation theory (Harter 1992) and cognitive evaluation theory (Deci and Ryan 1985) posit that individuals are more likely to manifest intrinsic motivation when they believe themselves to be more competent. Indeed, in an experimental study, Vallerand and Reid (1984) found that the effect of positive versus negative feedback on intrinsic motivation was mediated by perceived competence in a motor task. In another experimental study, Jussim et al. (1992, Study 3) found that positive versus negative feedback in an anagram task significantly affected intrinsic motivation through perceived competence. Similarly, Badami et al. (2011) found that positive versus negative feedback enhanced participants’ intrinsic motivation through perceived competence in a golf-putting task.

#### Effort intentions

Intrinsic motivation is commonly regarded as beneficial for learning (Stipek 2002; Guay et al. 2008). Research has shown that intrinsic motivation is associated with valued educational outcomes, such as challenge seeking (Boggiano et al. 1988; Vansteenkiste et al. 2004), persistence (Hardré and Reeve 2003; Vallerand and Bissonnette 1992), achievement (Grolnick et al. 1991; Miserandino 1996), and subjective well-being (Ryan and Connell 1989; Levesque et al. 2004). Intrinsic motivation is typically examined as a process variable, linking antecedents of motivation to outcome variables, including effort intentions. For example, in a meta-analysis of 21 articles in the context of physical education, Chatzisarantis et al. (2003) found that intrinsic motivation mediated the relationship between perceived competence and intentions to engage in physical exercise. Further, in a cross-sectional study into school drop-out among high school students, Vallerand et al. (1997) observed that self-determined motivation (a concept which includes intrinsic motivation) mediated the relation between perceived competence and intention to remain in school. In a similar study, Lavigne et al. (2007) found that self-determined motivation to study science mediated the relation between perceived competence and intention to pursue science education. Furthermore, in a cross-sectional study among teachers, Sørebø et al. (2009) reported a positive link between intrinsic motivation and intention to use e-learning facilities.

### Overview of the present studies

The present research adds to the extant literature on SRL strategies and motivation by examining the *causal effects* of strength-based versus deficit-based^1^ SRL strategies on students’ effort intentions, including the *mediating effects* of perceived competence and intrinsic motivation. We tested our research model (see Fig. 1) in two randomized experiments. In Study 1, we contrasted a strength-based SRL strategy with a deficit-based SRL strategy condition, and examined the effects on students’ perceived competence, intrinsic motivation, and effort intentions on a hypothetical school project. In Study 2, we added a neutral SRL strategy condition, and assessed the effects on students’ perceived competence, intrinsic motivation, and effort intentions on a professional development activity that they actually intended to carry out. *Hypothesis 1* was that strength-based versus deficit-based SRL strategies positively affect perceived competence, intrinsic motivation, and effort intentions. *Hypothesis 2* was that the effect of strength-based versus deficit-based SRL strategies on effort intentions is sequentially mediated by perceived competence and intrinsic motivation.

## Method Study 1

### Participants

The participants were 174 first-year to fourth-year bachelor’s students (32 % men), representing different schools, including Healthcare (*n* = 39), Management (*n* = 52), Education (*n* = 45), and Technology (*n* = 38). Ages ranged from 17 to 29, with a mean of 21.75 (*SD* = 2.64).

### Procedure

The students were recruited through an email, sent by their school, in which they were invited to take “a trial version of a new professional qualities test”, which would include completing a questionnaire. Those who accepted the invitation could start right away by clicking on a hyperlink. Participants first completed the “professional qualities test”, a 155-item inventory in which they were asked to indicate the extent to which 31 positive attributes applied to them. The test was based on the Dutch Abridged Big Five Circumplex (De Raad et al. 1992). Sample items are, “I do my work in an accurate manner” (accurate), “I often talk to a lot of people” (communicative), and “I am a dependable person” (dependable). Response categories ranged from 1 (*does not apply to me at all*) to 7 (*completely applies to me*). The scores on the five items of each subscale were averaged to calculate an index for each professional quality (all Cronbach’s alphas >.63). The test outcome showed a rank order of professional qualities, ranging from #1 (*applies most to me*) to #31 (*applies least to me*). After receiving the test outcome, participants were randomly assigned^2^ to a strength-based SRL strategy condition (*n* = 77) or a deficit-based SRL strategy condition (*n* = 97) in which they were instructed to select their #1 or their #31 ranked quality, respectively. Next, participants were asked to imagine that they signed up for a school project in which they could improve their #1 ranked professional quality (strength-based SRL strategy condition) or their #31 ranked professional quality (deficit-based SRL strategy condition), respectively. They then filled out the questionnaire. After completing the questionnaire, all participants were debriefed.^3^

### Measures

#### Manipulation checks

After being instructed to pick their #1 (strength-based SRL strategy condition) or their #31 (deficit-based SRL strategy condition) ranked quality, the participants were asked, “To what extent do you possess this professional quality?” Response categories ranged from 1 (*not at all*) to 9 (*completely*).

#### Perceived competence

Perceived competence was assessed using the Perceived Competence subscale of the Intrinsic Motivation Inventory (Ryan 1982). The items were slightly adjusted to refer to the project the participants had signed up for: (1) “I think I will be pretty good at this project”; (2) “Relative to other students, I think I will do pretty well at this project”; (3) “I feel pretty competent at this project”; (4) “I think I will be satisfied with my performance on this project”; (5) “I am pretty skilled at this project”; (6) “This is a project that I cannot do very well” (reverse scored). Response categories ranged from 1 (*completely disagree*) to 7 (*completely agree*). Items were averaged to create an index for perceived competence.

#### Intrinsic motivation

Intrinsic motivation was assessed using the Intrinsic Motivation subscales of the Academic Motivation Scale (AMS; Vallerand et al. 1992). The AMS contains three Intrinsic Motivation subscales of four items each. Following the procedure reported by others (e.g., Richer and Vallerand 1995; Vallerand 1997; Van Yperen and Hagedoorn 2003), we averaged the 12 items of the three subscales into one single indicator of intrinsic motivation. The general stem of the AMS, “Why do you go to school?” was adjusted to, “Why would you do this project?” A sample item is, “For the pleasure it gives me to know more about this project.” Response categories ranged from 1 (*not at all*) to 7 (*very much*). Intrinsic motivation was significantly related to perceived competence (*r* = .77, *p* < .001).

#### Effort intentions

Effort intentions were assessed using the following three-item scale: (1) “I intend to put effort into this project”; (2) “I am not going to do my best at this project” (reversed scored); (3) “I am determined to do this project”. Response categories ranged from 1 (*completely disagree)* to 7 (*completely agree)*. Items were averaged to create an index for effort intentions. Effort intentions were significantly related to perceived competence (*r* = .62, *p* < .001) and intrinsic motivation (*r* = .74, *p* < .001).

## Results Study 1

### Manipulation check

To check the manipulation, participants were asked to indicate to what extent they possessed the professional quality they had selected to improve. The results showed a highly significant difference, *M*_#1_ = 8.04 (*SD* = 1.21) versus *M*_#31_ = 2.47 (*SD* = 1.79), *F*(1, 161) = 528.80, *p* < .001, η^2^ = .77), allowing us to conclude that the manipulation was successful. That is, in the strength-based SRL strategy condition, participants selected a professional quality that they believed they possessed to a large degree (i.e., a perceived relative strength), whereas in the deficit-based SRL strategy condition participants selected a professional quality they believed they hardly possessed (i.e., a perceived relative shortcoming).

### Tests of Hypothesis 1

The means and standard deviations of the dependent variables by condition are presented in Table 1. *Hypothesis 1* posited that strength-based versus deficit-based SRL strategies positively affect perceived competence, intrinsic motivation, and effort intentions. Hence, we conducted a multivariate analysis of variance (MANOVA), with strength-based versus deficit-based SRL strategies as independent variable and perceived competence, intrinsic motivation, and effort intentions as dependent variables. The results yielded a highly significant overall effect,^4^ *F*(3, 170) = 71.27, *p* < .001, η^2^ = .56. Univariate analyses of variance (ANOVAs) revealed that, relative to students in the deficit-based SRL strategy condition, students in the strength-based SRL strategy condition were higher in perceived competence, *F*(1, 172) = 213.48, *p* < .001, η^2^ = .55, intrinsic motivation, *F*(1, 172) = 70.70, *p* < .001, η^2^ = .29, and effort intentions*, F*(1, 172) = 39.15, *p* < .001, η^2^ = .19. Thus, *Hypothesis 1* was empirically supported.

**Table 1:** Differences in means between strength-based self-regulated learning (SRL) strategies and deficit-based SRL strategies (Study 1)

	Strength-based SRL strategies (*n* = 77)		Deficit-based SRL strategies (*n* = 97)		*F*	*p* <	*η* ^2^
	*M*	*SD*	*M*	*SD*			
Perceived competence (Cronbach’s α = .96)	5.73	.81	3.32	1.26	213.48	.001	.55
Intrinsic motivation (Cronbach’s α = .95)	5.75	.74	4.41	1.23	70.70	.001	.29
Effort intentions (Cronbach’s α = .80)	5.93	.94	4.87	1.23	39.15	.001	.19

### Tests of Hypothesis 2

**Fig. 2:**
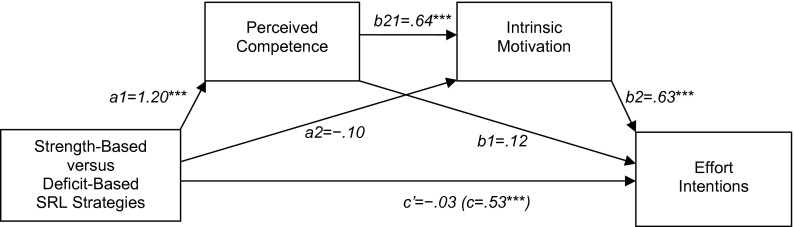
Multiple mediation model for the effect of strength-based versus deficit-based self-regulated learning (SRL) strategies on effort intentions (Study 1). *p < .05; **p < .01; ***p < .001

*Hypothesis 2,* positing that the effect of strength-based versus deficit-based SRL strategies on effort intentions was sequentially mediated by perceived competence and intrinsic motivation, was supported as well. We used Hayes’ (2013) PROCESS SPSS macro (model 6) to calculate the regression weights shown in Fig. 2. Model path estimates yielded a highly significant indirect path from strength-based versus deficit based SRL strategies, through perceived competence and intrinsic motivation, to effort intentions (a1 × b21 × b2); the direct path (c) was reduced to nonsignificant (c′) when the mediators were controlled for. Bootstrapping analysis, based on 5000 re-samples, showed a significant total indirect effect (a1 × b21 × b2 + a1 × b1 + a2 × b2) of point estimate .56 (95 % BCA-CI [.38, .74], *SE* = .09). Examination of the specific indirect effects revealed that neither the single effect through perceived competence (a1 × b1), point estimate = .14 (95 % BCA-CI [−.07, .35], *SE* = .11), nor the single effect through intrinsic motivation (a2 × b2), point estimate = −.06 (95 % BCA-CI [−.16, .05], *SE* = .05), was uniquely significant. Only the indirect path through both mediators (a1 × b21 × b2) was significant, point estimate = .48 (95 % BCA-CI [.33, .65], *SE* = .08), indicating that the effect of strength-based versus deficit-based SRL strategies on effort intentions was sequentially mediated by perceived competence and intrinsic motivation.

Finally, to test our research model (see Fig. 1) against alternative sequences of the mediating and dependent variables, we conducted structural equation modeling (SEM). The results revealed that, relative to the alternative sequences,^5^ the hypothesized sequence, *strength*-*based versus deficit*-*based SRL strategies* (S/D-SRL) → *perceived competence* (PC) → *intrinsic motivation* (IM) → *effort intentions* (EI), showed the best goodness of fit, *df* = 3, *x* ^2^ = 4.09, *p* = .25, CFI = 1.00, RMSEA = .05, PCFI = .50.

## Discussion Study 1

As expected, the findings of Study 1 showed that, relative to students who used deficit-based SRL strategies, students who used strength-based SRL strategies were higher in perceived competence, intrinsic motivation, and effort intentions. Note that the observed effect sizes were high compared to those typically found in feedback research (cf., Hattie and Timperley 2007). Furthermore, the results showed that the effect of strength-based versus deficit-based SRL strategies on effort intentions was sequentially mediated by perceived competence and intrinsic motivation.

However, a limitation of Study 1 was that we asked students to imagine a hypothetical project, rather than a professional development activity that they actually intended to carry out. Furthermore, in Study 1 we contrasted the two poles of the strength-based versus deficit-based SRL strategies dimension, so we do not know yet how intermediate strategies (e.g., neutral SRL strategies) affect students’ perceived competence, intrinsic motivation, and effort intentions. To address these issues, in Study 2, we asked students to think up and select a professional development activity that they actually intended to carry out. In addition, we included a neutral SRL strategy condition, in which participants aimed at improving a quality that they considered neither a strength nor a shortcoming.

## Method Study 2

### Participants

To replicate the findings of Study1 in a classroom setting, in Study 2 we invited the participants through their study skills teachers to conduct an assignment that was presented to them as “an exercise in talent development”. The participants were 267 first-year bachelor’s students (62 % men) from different schools of a Dutch university of applied sciences, including Healthcare (*n* = 75), Management (*n* = 49), Education (*n* = 46), and Technology (*n* = 97). Ages ranged from 17 to 28 years, with a mean of 19.78 (*SD* = 2.38). As Study 1 and Study 2 were conducted with a one-year interval, and Study 2 only included first-year students, no students participated in both studies.

### Procedure

The students were tested in groups of 5–25 participants. In 90-min sessions, the participants conducted a self-reflection and goal-selection exercise, and completed a questionnaire. The exercise was based on Seligman et al. (2005) and comprised the following five steps. First, using a Q-sorting procedure, students rank ordered 34 short descriptions of professional qualities, similar to those used in Study 1, on a scale ranging from #1 (*applies most to me*) to #34 (*applies least to me*). Second, participants were randomly assigned (see footnote 2) to a condition in which they were instructed to select one professional quality they wanted to work on during the following week, from their #1 to #5 ranked qualities (strength-based SRL strategy condition; *n* = 75), from their #15 to #19 ranked qualities (neutral SRL strategy condition; *n* = 90), or from their #30 to #34 ranked qualities (deficit-based SRL strategy condition; *n* = 102). Third, the participants described in their own words the professional quality they had chosen. Fourth, the participants listed as many activities as they could think of to improve themselves on this quality. Table 2 shows a number of examples of the activities that the participants thought up. Fifth, the participants selected from the activities they had listed, one activity to carry out during the following week. Next, the participants responded to the dependent variables and the manipulation check. Finally, the participants were debriefed (see footnote 3).

**Table 2:** Examples of professional development activities that students thought up themselves, Study 2

Professional quality	Professional development activity
Creative	To draw a sketch every time a have an good idea
Decisive	To take the lead in our next workgroup meeting
Disciplined	To make a work plan each morning
Driven	To attend extra-curricular lectures
Focussed	To make sure that we finish our project this week
Independent	To work alone on our project for one day, to get it back on track
Initiative	To recruit new clients at my job
Leadership	To observe others how they chair a meeting
Optimistic	To list the positive attributes of all of my project group members
Responsible	To fulfil every commitment that I make during the next week
Sociable	To invite others to work on our assignment together
Unprejudiced	To chat with class mates that I usually don’t talk to

### Measures

#### Manipulation check

Participants were asked to indicate the following: “I have chosen to develop a professional quality that I am …”: (1) “good at”; (2) “neither good nor bad at”; (3) “not good at.”

#### Dependent variables

The three dependent variables were assessed using the same scales as in Study 1. However, in the wording of the items, “this project” was replaced by “this activity”. Intrinsic motivation was significantly related to perceived competence (*r* = .56, *p* < .001). Effort intentions were significantly related to perceived competence (*r* = .37, *p* < .001) and intrinsic motivation (*r* = .73, *p* < .001).

## Results Study 2

### Manipulation check

In response to the item “I have chosen to develop a professional quality that I am …”: (1) “good at”; (2) “neither good nor bad at”; (3) “not good at”, almost all participants (86.89 %) picked the option that matched the condition they were assigned to (Cramér’s V = .81; *p* < .001). We therefore concluded that the manipulation was successful. That is, the participants in the strength-based SRL strategy condition identified a professional quality they believed they were good at (i.e., a perceived relative strength), the participants in the neutral SRL strategy condition identified a professional quality they believed they were neither good nor bad at, and the participants in the deficit-based SRL strategy condition identified a professional quality they believed they were bad at (i.e., a perceived relative shortcoming).

### Tests of Hypothesis 1

The means and standard deviations of the dependent variables by condition are shown in Table 3. In line with *Hypothesis 1,* multivariate analysis of variance (MANOVA) yielded a significant overall effect of SRL strategy condition on the dependent variables,^6^ *F*(6, 526) = 5.58, *p* < .001, η^2^ = .06. Univariate analyses of variance (ANOVAs) revealed that the strength-based versus deficit-based SRL strategy manipulation significantly affected perceived competence, *F*(2, 264) = 17.55, *p* < .001, η^2^ = .12, intrinsic motivation, *F*(2, 264) = 6.00, *p* < .01, η^2^ = .04, and effort intentions, *F*(2, 264) = 3.60*, p* < .05, η^2^ = .03. As indicated by the different superscripts in Table 3 (*p* < .05 at the minimum), post hoc analyses revealed that, relative to participants in the deficit-based SRL strategy condition, participants in the strength-based SRL strategy condition were significantly higher in perceived competence, intrinsic motivation, and effort intentions; this is a perfect replication of the findings of Study 1. Furthermore, relative to participants in the deficit-based SRL strategy condition, participants in the neutral SRL strategy condition were significantly higher in perceived competence, and relative to the neutral SRL strategy condition, participants in the strength-based SRL strategy condition were significantly higher in perceived competence and intrinsic motivation.

**Table 3:** Differences in means between strength-based self-regulated learning (SRL) strategies, neutral SRL strategies, and deficit-based SRL strategies (Study 2)

	Strength-based SRL strategies (*n* = 75)		Neutral SRL strategies (*n* = 90)		Deficit-based SRL strategies (*n* = 102)		*F*	*p* <	*η* ^2^
	*M*	*SD*	*M*	*SD*	*M*	*SD*			
Perceived competence (Cronbach’s α = .90)	5.29^a^	.94	4.59^b^	1.29	4.26^c^	1.18	17.55	.001	.12
Intrinsic motivation (Cronbach’s α = .95)	4.92^a^	1.10	4.39^b^	1.46	4.16^b^	1.67	6.00	.01	.04
Effort intentions (Cronbach’s α = .85)	5.44^a^	1.18	5.06^ab^	1.59	4.83^b^	1.66	3.60	.05	.03

W*ithin each row, different superscripts* indicate significant group differences at level *p* < .05

### Tests of Hypothesis 2

Study 2 also yielded additional empirical support for *Hypothesis 2*. To test this hypothesis, we first recoded the independent variable into two dummy variables. The deficit-based SRL strategy condition, representing common practice, was used as the reference group. Thus, Dummy 1 was used to compare the strength-based SRL strategy condition with the deficit-based SRL strategy condition, and Dummy 2 was used to compare the neutral SRL strategy condition with the deficit-based SRL strategy condition.

**Fig. 3:**
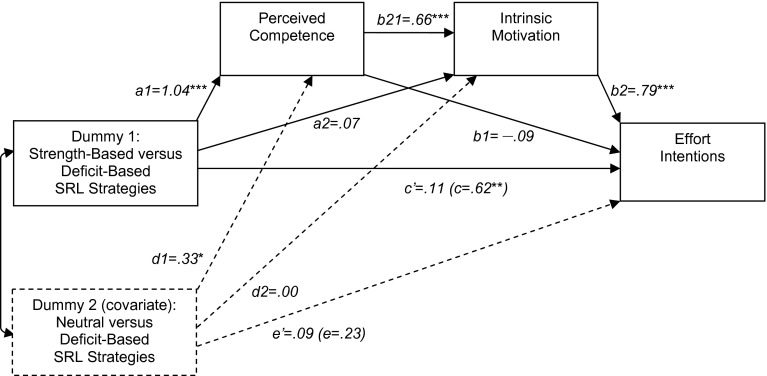
Multiple mediation model for the effect of strength-based versus deficit-based self-regulated learning (SRL) strategies on effort intentions (Study 2). * p < .05;** p < .01; *** p < .001

In the first analysis, we treated Dummy 1 as the primary independent variable and Dummy 2 as a covariate. Figure 3 displays the path estimates obtained using Hayes’ (2013) PROCESS SPSS macro (model 6). The results showed a highly significant indirect path through perceived competence and intrinsic motivation (a1 × b21 × b2). The direct path (c) was reduced to nonsignificant (c’) when the mediators were controlled for. Bootstrapping analysis, based on 5000 re-samples, yielded a significant total indirect effect (a1 × b21 × b2 + a1 × b1 + a2 × b2), point estimate = .51 (95 % BCA-CI [.19, .86], *SE* = .17). The indirect path through both mediators (a1 × b21 × b2) was significant, point estimate = .54 (95 % BCA-CI [.34, .78], *SE* = .11), whereas the single indirect paths, through perceived competence (a1 × b1), point estimate = −.09 (95 % BCA-CI [−.25, .06], *SE* = .08), and intrinsic motivation (a2 × b2), point estimate = .06 (95 % BCA-CI [−.26,. 38], *SE* = .16), were not significant.

In the second analysis, we treated Dummy 2 as the primary independent variable and Dummy 1 as a covariate. The analysis yielded a nonsignificant effect on effort intentions, which is consistent with the results of post hoc analysis, indicating a nonsignificant difference in effort intentions between the deficit-based SRL strategy condition and the neutral SRL strategy condition.

Finally, structural equation modeling (SEM) analysis revealed an excellent goodness of fit for the hypothesized sequence, *strength*-*based versus deficit*-*based SRL strategies* (S/D-SRL) → *perceived competence* (PC) → *intrinsic motivation* (IM) → *effort intentions* (EI), *df* = 5, *x* ^2^ = 2.08, *p* = .84, CFI = 1.00, RMSEA = .00, PCFI = .50, whereas the ratios of the alternative models were below threshold level.^7^

## Discussion Study 2

In line with the findings of Study 1, the results of Study 2 show that, relative to deficit-based SRL strategies, strength-based SRL strategies lead to higher perceived competence, intrinsic motivation, and effort intentions. Furthermore, Study 2 yielded additional empirical support for the hypothesis that the effect of strength-based versus deficit-based SRL strategies on effort intentions is subsequently mediated by perceived competence and intrinsic motivation. The findings of Study 2 extend those of Study 1 by demonstrating that these effects hold under real-life conditions, that is, when students themselves think up and select a professional development activity that they actually intend to carry out, rather than imagine a hypothetical project. In addition, the findings of Study 2 show that strength-based SRL strategies lead to more optimal outcomes than neutral SRL strategies. That is, strength-based SRL strategies resulted in higher perceived competence and intrinsic motivation; the difference in effort intentions was in the expected direction, but not significant.

Notably, the effect sizes in Study 2 were smaller than in Study 1. This may be explained by two differences in methodology. First, in Study 1 the students picked *the highest* ranked versus *the lowest* ranked professional quality to work on, whereas in Study 2, the students picked *one of the five highest* ranked, versus *one of the five lowest* ranked qualities to work on. Second, in Study 1 the students selected a hypothetical project, whereas in Study 2 the students selected a concrete activity that they had thought up themselves. Both differences in methodology are likely to diminish the contrast between the strength-based and the deficit-based SRL strategy condition.

## General discussion

On the basis of our consistent findings across two randomized experiments, we conclude that, relative to deficit-based SRL strategies, strength-based SRL strategies positively affect students’ perceived competence, intrinsic motivation, and effort intentions. Moreover, in both studies, we found that perceived competence and intrinsic motivation sequentially mediated the effect of strength-based versus deficit-based SRL strategies on effort intentions.

These findings extend previous research in several ways. First, because we used a randomized experimental design, we were able to establish a causal relation between strength-based versus deficit-based SRL strategies and the dependent variables. As we used specific, unidimensional interventions, we are confident that the observed effects can be attributed to the use of strength-based versus deficit-based SRL strategies, that is, the selection of a project (Study 1) or a self-thought up activity (Study 2) to improve a perceived relative strength versus shortcoming. Second, because we contrasted strength-based SRL strategies with deficit-based SRL strategies, we now know that strength-based SRL strategies lead to higher perceived competence, intrinsic motivation, and effort intentions than the common deficit-based SRL strategies. We also demonstrated that strength-based SRL strategies lead to higher perceived competence and intrinsic motivation relative to neutral SRL strategies (i.e., a condition in which students selected an activity to improve a quality that they perceived as neither a strength nor a shortcoming). Third, the results of our multi-mediator analysis and structural equation modeling provide a better understanding of *why* strength-based versus deficit-based SRL strategies differently affect students’ effort intentions: namely, through perceived competence and intrinsic motivation.

More in general, our findings add to the literature on enhancing students’ intrinsic motivation to learn. Several theories, such as effectance motivation theory (Harter 1992), self-determination theory (Ryan and Deci 2000), and self-concordance theory (Sheldon and Elliot 1999), posit that intrinsic motivation is beneficial for learning. However, these theories do not articulate *how* students can self-select intrinsically motivating activities to improve their competencies. The present research demonstrates that students can self-select intrinsically motivating professional development activities by identifying their perceived relative strengths and aiming at further improving those strengths.

It is important to note, however, that our findings seem to contradict the position of scholars who posit that self-enhancing interventions do not improve learning (Forsyth et al. 2007; cf. Baumeister et al. 2003; Mueller and Dweck 1998). Specifically, Forsyth et al. (2007) suggested on the basis of experimental research that self-enhancing interventions may even be detrimental to learning. In their study, Forsyth et al. (2007) manipulated the feedback that students received while preparing for a psychology exam. Their results indicated that students who received self-bolstering feedback performed worse relative to a control group. However, three specific differences between Forsyth et al. (2007) and our research may explain these divergent findings. First, Forsyth et al. (2007) conducted a feedback intervention which affected students’ efforts while they were working toward a preset goal, whereas we conducted a goal-selection intervention that affected which activities the students selected. As explained by Vancouver et al. (2002), self-enhancing interventions may differently affect students’ learning, depending on the self-regulatory process that is affected (e.g., performance monitoring versus goal-selection). Second, Forsyth et al. (2007) affirmed students on the global level of self-esteem by sending emails with statements such as, “Hold your head and your self-esteem high”. In contrast, our strength-based SRL strategy intervention affirmed students on specific professional qualities, such as “creative”, “focused”, or “unprejudiced”. Indeed, research has shown that the effects of affirmative interventions may differ depending on the level of specificity of the message (Hattie and Timperley 2007; Baumeister et al. 2003). Third, the study of Forsyth et al. (2007) was conducted in the context of the mandatory curriculum, whereas our research was conducted in the context of professional self-development. Clearly, when preparing for a mandatory exam, intrinsic motivation is less of a prerequisite for effort (Sansone and Smith 2000; Lepper and Henderlong 2000), however, in the context of professional self-development, intrinsic motivation is crucial for ensuring effort. In sum, the phase of the self-regulatory process (performance-monitoring versus goal-selection), the level of specificity (global self-esteem versus specific qualities), and the amount of autonomy (externally controlled versus self-development) may be significant moderators of the effects of self-enhancing interventions on students’ efforts. As far as we are concerned, testing the moderating role of these factors should be put high on the empirical agenda.

### Strengths and limitations

The consistency of the findings across both experimental studies indicates the robustness of our findings. In addition, because we tested the effects of SRL strategies under field conditions, the ecological validity and practical relevance of our studies is high, which is an important strength. In contrast, the reliance on self-report measures, albeit appropriate for studies on motivational processes, may be considered a limitation. However, the assessment of self-report effort intentions, rather than actual behavioral effort, is an obvious consequence of the methodology we used. That is, the consequence of using Seligman et al.’s (2005) procedure in a field setting is that students themselves can think up a wide range of different professional development activities. As can be seen in Table 2, these activities vary substantially in terms of time expenditure. Consequently, these activities are not comparable at the behavioral level.

Furthermore, although our findings provide empirical evidence for the causal effects of strength-based versus deficit-based SRL strategies on perceived competence, intrinsic motivation, and effort intentions, our follow-up mediation and SEM analyses only provide suggestive evidence that the effects of these SRL strategies on effort intentions are sequentially mediated by perceived competence and intrinsic motivation. In future studies, series of experiments may be conducted to empirically establish the proposed causal chain (e.g., Spencer et al. 2005).

### Practical implications

Our findings have clear implications for the use of SRL strategies in higher professional and vocational education. Many educators aim for their students to become self-regulating learners who are driven to work on their professional development. However, the question is whether deficit-based SRL strategies, which are common practice, are the most optimal way to motivate students to put effort into professional development activities. Professional self-development requires willingness to expend effort, which appears to be a function of perceived competence and intrinsic motivation. Our findings demonstrate that these outcomes are induced by strength-based rather than by deficit-based SRL strategies. Therefore, we suggest that, to stimulate students’ to put effort into professional development activities, educators may teach their students to use strength-based SRL strategies. For example, the strength-based SRL strategy that we examined in Study 2 may be taught in mentoring, tutoring or study skills classes. For more practical suggestions, see Bouskila-Yam and Kluger (2011), Clifton and Anderson (2002), and Linley (2008).

Finally, just to be clear, we do not suggest that strength-based SRL strategies are *a substitute* for deficit-based SRL strategies. Deficit-based SRL strategies are a sine qua non to qualify for any profession. That is, students need to work on diminishing the gap between their present level of competency and the prevailing standards for a particular profession. However, to enhance students’ motivation to put effort into professional self-development activities, strength-based SRL strategies may make a valuable complement to the common deficit-based SRL strategies.

## Footnotes

1 We conducted our studies at a university of applied sciences that practices competency-based education. In mentoring, tutoring, and study skills classes, students reflect on their shortcomings relative to the standards explicated in a competency profile. Based on this reflection, students set their goals and select their elective courses and projects for the next semester. Thus, the deficit-based SRL strategy condition reflects common practice.

2 Sample sizes are not equal across the conditions due to the unrestricted random assignment procedure used (Survey Monkey^®^).

3 In the debriefing, we explained the aims and expectations of our research to the students. We proposed that diminishing shortcomings is indispensable for mastering a profession, but that developing strengths might make a valuable complement to their professional development. Accordingly, we suggested that all students work both on improving their shortcomings and on further improving their strengths during their education.

4 No significant interaction effect between sex and strength-based versus deficit-based SRL strategies on the dependent variables was found, *F*(3, 168) = 1.88, *p* = .36.

5 S/D-SRL → PC → EI → IM (*df* = 3, *x* ^2^ = 75.58, *p* = .00, CFI = .83, RMSEA = .37, PCFI = .42) S/D-SRL → IM → PC → EI (*df* = 3, *x* ^2^ = 134.6, *p* = .00, CFI = .69, RMSEA = .50, PCFI = .35) S/D-SRL → IM → EI → PC (*df* = 3, *x* ^2^ = 51.87, *p* = .00, CFI = .65, RMSEA = .54, PCFI = .32) S/D-SRL → EI → PC → IM (*df* = 3, *x* ^2^ = 158.70, *p* = .00, CFI = .64, RMSEA = .55, PCFI = .32) S/D-SRL → EI → IM → PC (*df* = 3, *x* ^2^ = 36.07, *p* = .00, CFI = .76, RMSEA = .45, PCFI = .38).

6 No significant interaction effect between sex and strength-based versus deficit-based SRL strategies on the dependent variables was found, *F*(6, 520) = .55, *p* = .77.

7 S/D-SRL → PC → EI → IM (*df* = 5, *x* ^2^ = 62.10, *p* = .00, CFI = .85, RMSEA = .21, PCFI = .43) S/D-SRL → IM → PC → EI (*df* = 5, *x* ^2^ = 189.78, *p* = .00, CFI = .52, RMSEA = .37, PCFI = .26) S/D-SRL → IM → EI → PC (*df* = 5, *x* ^2^ = 83.47, *p* = .00, CFI = .78, RMSEA = .24, PCFI = .40) S/D-SRL → EI → PC → IM (*df* = 5, *x* ^2^ = 194.45, *p* = .00, CFI = .51, RMSEA = .38, PCFI = .26) S/D-SRL → EI → IM → PC (*df* = 5, *x* ^2^ = 28.12, *p* = .00, CFI = .94, RMSEA = .13, PCFI = .47).
